# Vitreous Incarceration in Patients Undergoing Second 20-Gauge Pars Plana Vitrectomy for Recurrent Retinal Detachment

**DOI:** 10.5402/2011/456191

**Published:** 2011-10-09

**Authors:** Yongxin Zheng, Haotian Lin, Wen Liu, Dandan Wang, Suying Huang

**Affiliations:** State Key Laboratory of Ophthalmology, Zhongshan Ophthalmic Center, Sun Yat-Sen University, Guangzhou 510060, China

## Abstract

*Purpose*. To observe and classify vitreous incarcerations in patients undergoing second 20-gauge pars plana vitrectomy (PPV) for recurrent retinal detachment. *Methods*. Retrospective noncomparative consecutive case series. Eighty-two consecutive patients with recurrent retinal detachment were included. The previous sclerotomy sites were examined by our sclera depression method and the vitreous incarceration were classified into Grade 0–IV by their severity under surgical microscope before second surgery. The relationship of vitreous incarceration and different ports was statistically investigated in our included patients. *Results*. Vitreous incarceration in the previous sclerotomy sites were found frequently. Vitreous cutter sites were most involved, but the infusion pipe sites were the least. According to our classification and definition, Grade III and IV of vitreous incarceration in all the three different sclerotomy sites accounted for 32.5%. Grade II of vitreous incarceration consisted of 12.6%. Grade 0 and I in all the three different sclerotomy sites were 54.8%. The frequency of all grades of vitreous incarceration in light port or vitreous cutter port was significant higher than that in infusion port. *Conclusions*. Vitreous incarceration in light port and vitreous cutter port are found more common than in infusion port for 20-gauge PPV with our new method.

## 1. Introduction

The causes of vitreous surgery failure have been a controversial issue for the century [1–3]. It was considered that anterior proliferative vitreoretinopathy (APVR) is the main reason of the initial or repeated vitreous surgeries [1, 2]. However, APVR was significantly associated with residual basal vitreous and could be prevented effectively by its complete removal during PPV [3]. Furthermore, many pathological studies have demonstrated that APVR was related to vitreous incarceration of sclerotomy sites [4–6]. Unfortunately, it is difficult to inspect the inner aspect of sclerotomy sites with conventional techniques, especially when there is massive residual vitreous, vitreous opacity, or hemorrhage [6]. Therefore, we developed a practical sclera depression method to examine the previous sclerotomy sites under surgical microscope before second 20-gauge PPV and defined a new method for classification of vitreous incarcerations in sclerotomy sites. Then we investigated the relationship of severity of vitreous incarcerations and different 20-gauge sclera port in our study group.

## 2. Materials and Methods

### 2.1. Included Cases

82 consecutive hospitalized patients (82 eyes) with recurrent retinal detachment, who were planned to receive second 20-gauge PPV from 7 provinces in southern China during January 2000 to December 2007, were included in our study. Clinical information of included patients was summarized in Table 1.

None of the 82 patients had APVR prior to the initial vitrectomy. The previous vitreous surgeries were all standard 20-gauge three-port PPV. All cases were rhegmatogenous retinal detachment (RD) at this recurrence. For the study, institutional review board/ethics committee approval was not required.

### 2.2. Surgery and Observation Methods

A standard three-port PPV was performed on all the 82 patients by the same experienced surgeon. The main surgical procedures were similar to the methods described by Lewis and Aabarg [3]. However, our surgery and observation methods were special, as follows. New sclerotomy sites were chosen beside the previous ones (all were with 20-gauge sclerotomies). After sclerotomies, the inner aspects of the previous sclerotomy sites were carefully examined by our sclera depression method [10] before vitrectomy. The ports for vitreous cutter and light pipe should be exchanged for observation of the previous sites near each other. In phakic eyes, when a 45° prismatic lens was placed and the slant was faced to the new sclerotomy site, the assistor should help indenting the sclera behind the site. Then the surgeon inserted a illuminative lighter shallowly at another port to visualize the inner aspect around the new sclerotomy site. But in aphakic eye, we did not use any contact prismatic lens. The surgeon just held a sclera depressor to indent the sclera behind the former sclerotomy site and inserted the illuminative pipe through another port to visualize the area of indented sclera around one former sclerotomy site under the surgical microscopy.

### 2.3. Classification of Vitreous Incarceration in Sclerotomy Sites

**Figure 1:**
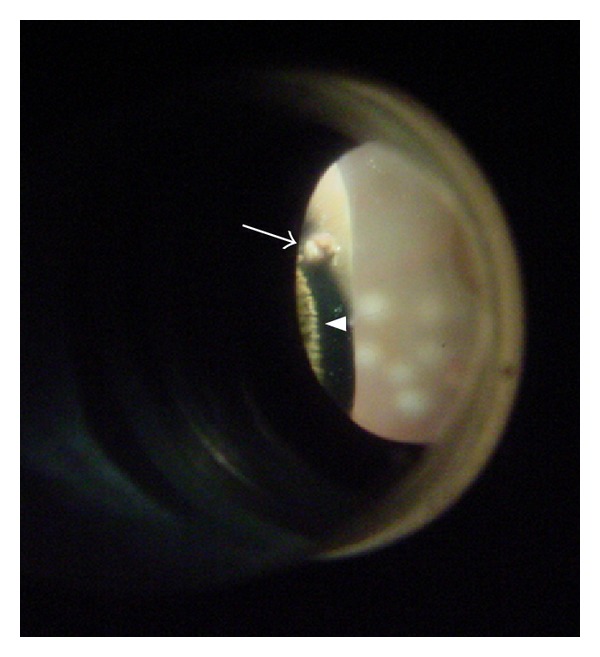
Photograph of inner aspect of sclerotomy under sclera depression. At 10 o'clock, the pars plicate can be seen (arrow head) and the sclerotomy appeared white injury without vitreous incarceration (arrow), which is classified as Grade 0.

**Figure 2:**
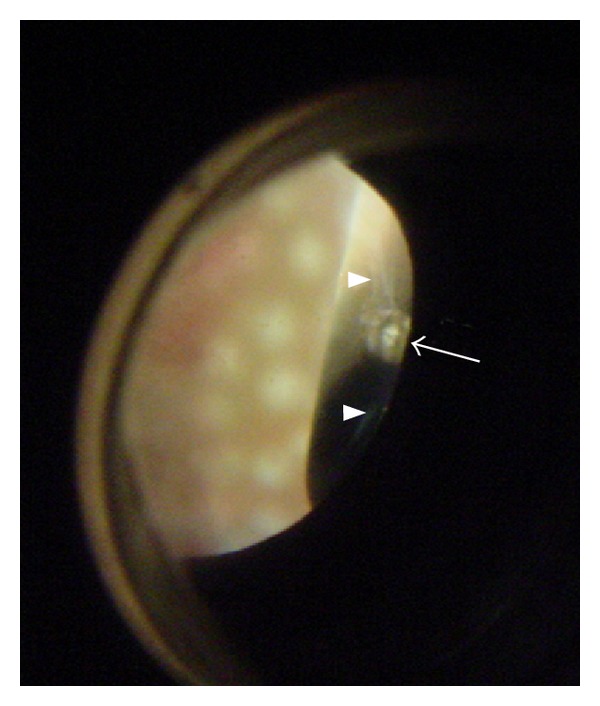
Photograph of inner aspect of sclerotomy under sclera depression. At 2 o'clock, the inner aspect of the sclerotomy presents a round white gap (arrow). The white vitreous fibers are incarcerated in the incision (arrow head) which is classified as Grade I.

**Figure 3:**
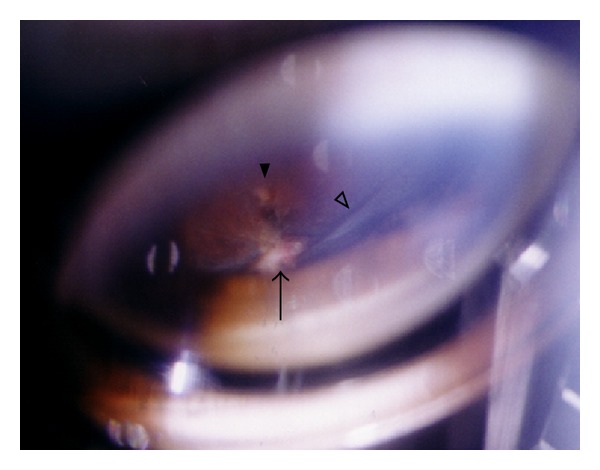
Photograph of sclerotomy-related complication. An amount of vitreous (open arrow head) is incarcerated in the inner incision at 10 o'clock (arrow), which pulls retina leading atrophic retinal hole (arrow head). It is classified as Grade II.

**Figure 4:**
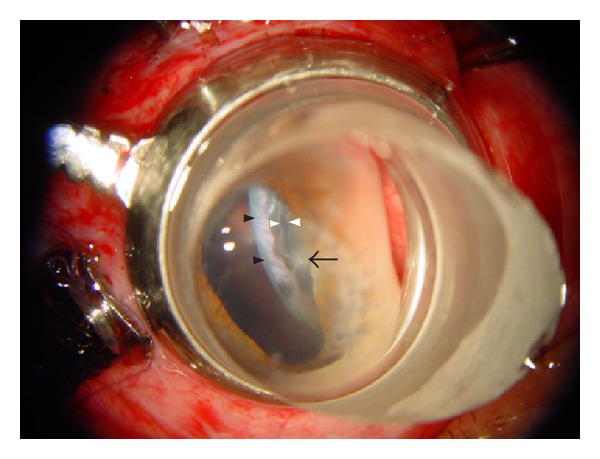
A total avulsion of the vitreous base occurred in the ora serrata posterior to the previous incision at 2 o'clock, which exposed the radial red and white lines like zebrine striations (black arrow head). The avulsed ciliary epithelium curled on pars plana (white arrow head). The incarcerated vitreous in the sclerotomy has been fibrosis (black arrow). It is classified as Grade III.

**Figure 5:**
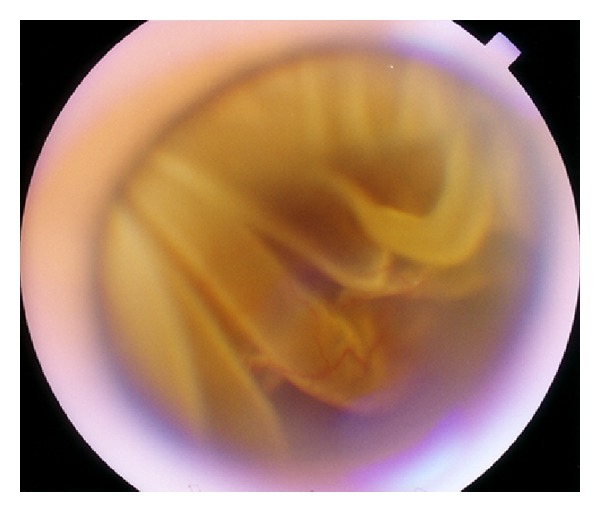
The patient underwent an unsuccessful vitrectomy 13 days before. When he was examined, the retina was incarcerated in both superior sclerotomies. This figure represents Grade IV of retinal incarceration in sclerotomy site.

The vitreous incarceration in the inner aspect of sclerotomy sites were classified as five grades by their severity. Grade 0 was no vitreous incarceration (Figure 1); Grade I had visible vitreous incarceration without traction on the retina (Figure 2); Grade II had vitreous incarceration with traction on the retina causing retinal detachment or trough (Figure 3); Grade III was vitreous incarceration leading to retinal breaks (Figure 4); Grade IV had retinal incarceration in sclerotomy site (Figure 5).

### 2.4. Analysis and Statistics

The chi-square test was used to compare a possible difference in the frequencies of vitreous incarceration and different sclera port.

## 3. Results

There were 246 previous sclerotomy sites (one eye with three different ports) observed in the 82 included recurrent retinal detachment patients (82 eyes), of which 164 (66.7%) previous sclerotomy sites were found with different degrees of vitreous incarceration. 

According to our classification and definition, there were 80 sites (32.5%) with vitreous incarceration of Grade III and IV. Grade II of vitreous incarceration were found in 31 previous sclerotomy sites (12.6%). There were 135 sclerotomy sites (54.8%) classified as Grade 0 and I. The frequencies of vitreous incarcerations of the five defined grades at the three different ports of sclerotomy sites in the 82 included eyes are summarized in Table 2.

To compare the frequencies of vitreous incarceration in different sclerotomy sites of standard three-port pars plana vitrectomy, only 12 patients (14.6%) were found without vitreous incarceration in the vitreous cutter sites, and 14 patients (17.1%) were without vitreous incarceration in the light pipe sites, but the infusion pipe sites in 56 patients (68.3%) were free of vitreous incarceration. We could see that the least sclerotomy-related complications occurred in infusion port, but the retinal breaks caused by sclerotomy-related complications were the most frequency in vitreous cutter port. However, compared with two groups in light port and vitreous cutter port, their difference was no significant (*χ* ^2^ = 3.178, *P* = 0.529). The frequency of all grades of light port (82.9%) or vitreous cutter port (85.4%) was significant higher than that of infusion port (31.7%; *χ* ^2^ = 36.536, *P* < 0.001, and *χ* ^2^ = 41.759, *P* < 0.001).

## 4. Discussion

Many studied have shown that the vitreous incarceration in sclerotomy sites inducing APVR, were the main reason for recurrent retinal detachment after 20-gauge PPV [11–15]. However, with conventional clinical methods such as indirect ophthalmoscope or three-mirror biomicroscopy with traditional sclera depression [6], it is extremely hard to observe the inner aspect of sclerotomy sites in detail, so the peripheral retina or the vitreous base is often ignored or only examined roughly [16–18]. It would be much more difficult to observe these areas in case of vitreous hemorrhage, vitreous opacity, or opaque vitreous skirt [6]. Although ultrasound biomicroscopy (UBM) have been used for detecting the inner aspect of sclerotomy sites before surgery [12, 13], by which the degree of vitreous incarceration could be graded indirectly [13, 14]. However, UBM could not identify different tissues incarcerated in sclerotomy sites due to the same echo of retina and vitreous. Therefore, a practical method should be developed or vitreous incarceration would be continued to be ignored and APVR could not be effectively prevented. In our study, we examine and classify the vitreous incarceration in the inner aspect of previous 20-gauge PPV sclerotomy sites under the surgical microscopy with our sclera depression method intraoperatively [10]. Our study demonstrated that this new clinical method provides a simple and feasible procedure. 

On the basis of our definition and classification, we found that vitreous incarceration of Grade III and IV together involved 32.5% of previous 20-gauge sclerotomy sites, and vitreous incarceration of Grade II involved 12.6% of previous 20-gauge sclerotomy sites. What should deserve our greatest attention is that the frequency of all grades of light port (82.9%) or vitreous cutter port (85.4%) was significant higher than that of infusion port (31.7%; *χ* ^2^ = 36.536, *P* < 0.001 and *χ* ^2^ = 41.759, *P* < 0.001). It means that after 20-gauge PPV, vitreous incarceration in light port and vitreous cutter port is more common than in infusion port, and the ports for vitreous cutter and light pipe should be exchanged for observation of the previous sites around each other and completely vitrectomy surrounding the two ports. 

In conclusion, vitreous incarceration in previous sclerotomy sites can be observed and classified with our new method in patients undergoing second 20-gauge PPV for recurrent retinal detachment. Vitreous incarceration in light port and vitreous cutter port is more common than in infusion port for the standard 20-gauge PPV. The most effective way to prevent the sclerotomy-related complications is to remove vitreous at basal and pars plana area completely during initial 20-gauge PPV, especially attention should be paid for the basal part surrounding light port and vitreous cutter port.

## Figures and Tables

**Table 1:** Clinical information of 82 patients included in this study.

Patient variables	*N*	%
Age (mean 32 years)		
≤32	40	49
>32	42	51
Gender		
Male	66	80
Female	16	20
Causal diseases [7]		
Rhegmatogenous retinal detachment	38	46
Ocular trauma	34	42
Branch retinal vein occlusion	3	4
Suppurative endophthalmitis	3	4
Proliferative diabetic vitreoretinopathy	2	2
Acute retinal necrosis syndrome	2	2
Standard classifications of eye trauma		
Open-global injury	29	35
Closed-global injury	5	6
Number of prior vitrectomies		
1	57	70
2	21	26
3	4	4
Other medical records		
Encircling	54	66
Silicone oil tamponade	33	40
Lens status at this time		
Phakia	31	38
Aphakia	37	45
Pseudophakia	14	17
PVR* [8, 9]		
A	3	4
B	19	23
C1–C3	43	52
D1–D3	17	21
APVR^§^		
Circumferential contraction	3	4
Anterior displacement	35	43
Circumferential contraction and anterior displacement	18	22
First PPV—this PPV interval (month)		
≤3	38	46
>3	44	54

*PVR: proliferative vitreoretinopathy; ^§^APVR: anterior PVR.

**Table 2:** Frequency of vitreous incarceration of different grades at each sclerotomy sites in the 82 included eyes.

Different sites	Grade 0		Grade I		Grade II		Grade III		Grade IV		Total	
	*N*	%	*N*	%	*N*	%	*N*	%	*N*	%	*N*	%
Infusion pipe	56	68.3	9	11.0	13	15.9	4	4.9	0	0	26	31.7
Light pipe	14	17.1	28	34.1	9	11.0	10	12.2	21	25.6	68	82.9
Vitreous cutter	12	14.6	16	19.5	9	11.0	9	11.0	36	43.9	70	85.4

Total	82	33.3	53	21.5	31	12.6	23	9.3	57	23.2	164	66.7
